# Canine cancer immunotherapy studies: linking mouse and human

**DOI:** 10.1186/s40425-016-0200-7

**Published:** 2016-12-20

**Authors:** Jiwon S. Park, Sita S. Withers, Jaime F. Modiano, Michael S. Kent, Mingyi Chen, Jesus I. Luna, William T. N. Culp, Ellen E. Sparger, Robert B. Rebhun, Arta M. Monjazeb, William J. Murphy, Robert J. Canter

**Affiliations:** 1 Department of Surgery, University of California Davis Medical Center, Sacramento, CA 95817 USA; 2 Department of Surgical and Radiological Sciences, School of Veterinary Medicine, University of California-Davis, Davis, CA 95616 USA; 3 Department of Veterinary Clinical Sciences, College of Veterinary Medicine, Animal Cancer Care and Research Center, Center for Immunology, Masonic Cancer Center, and Stem Cell Institute, University of Minnesota, St. Paul, MN 55108 USA; 4 The Center for Companion Animal Health, Department of Surgical and Radiological Sciences, School of Veterinary Medicine, University of California-Davis, Davis, CA 95616, USA; 5 Department of Pathology and Laboratory Medicine, University of California Davis Medical Center, Sacramento, CA 95817 USA; 6 Laboratory of Cancer Immunology, Department of Dermatology, University of California Davis Medical Center, Sacramento, CA 95817 USA; 7 Department of Veterinary Medicine and Epidemiology, School of Veterinary Medicine, University of California Davis, Davis, CA 95616 USA; 8 Department of Radiation Oncology, University of California Davis Medical Center, Sacramento, CA 95817 USA; 9 Dermatology and Internal Medicine, University of California Davis Medical Center, Sacramento, CA 95817 USA; 10 Department of Surgery, Division of Surgical Oncology, University of California Davis Medical Center, Sacramento, CA 95817 USA; 11 Department of Dermatology, Department of Internal Medicine, Division of Hematology/Oncology, School of Medicine, University of California, Davis, USA

**Keywords:** Canine model, Cancer immunotherapy, Spontaneous cancer, Outbred, Murine model

## Abstract

Despite recent major clinical breakthroughs in human cancer immunotherapy including the use of checkpoint inhibitors and engineered T cells, important challenges remain, including determining the sub-populations of patients who will respond and who will experience at times significant toxicities. Although advances in cancer immunotherapy depend on preclinical testing, the majority of in-vivo testing currently relies on genetically identical inbred mouse models which, while offering critical insights regarding efficacy and mechanism of action, also vastly underrepresent the heterogeneity and complex interplay of human immune cells and cancers. Additionally, laboratory mice uncommonly develop spontaneous tumors, are housed under specific-pathogen free conditions which markedly impacts immune development, and incompletely model key aspects of the tumor/immune microenvironment. The canine model represents a powerful tool in cancer immunotherapy research as an important link between murine models and human clinical studies. Dogs represent an attractive outbred combination of companion animals that experience spontaneous cancer development in the setting of an intact immune system. This allows for study of complex immune interactions during the course of treatment while also directly addressing long-term efficacy and toxicity of cancer immunotherapies. However, immune dissection requires access to robust and validated immune assays and reagents as well as appropriate numbers for statistical evaluation. Canine studies will need further optimization of these important mechanistic tools for this model to fulfill its promise as a model for immunotherapy. This review aims to discuss the canine model in the context of existing preclinical cancer immunotherapy models to evaluate both its advantages and limitations, as well as highlighting its growth as a powerful tool in the burgeoning field of both human and veterinary immunotherapy.

## Background

The ability of the immune system to recognize and eradicate transformed cells is the central rationale behind the application of immunotherapy for cancer [1]. Recent breakthrough developments in cancer immunotherapy include checkpoint blockade therapy targeting cytotoxic T-lymphocyte-associated antigen 4 (CTLA-4) and programmed death receptor-1 (PD-1) as well as adoptive transfer of engineered T cells or chimeric antigen receptor (CAR) T cells [2–9]. Yet, despite the exciting success of these therapies, only a fraction of patients durably responds to treatment. Hence, a critical issue for the clinical translation of cancer immunotherapy is determining factors predictive of response, and unlike traditional chemotherapy or targeted therapy, key aspects of the patient’s immune milieu are likely to be as important as tumor-related factors in determining response and toxicity.

Data from experiments in mouse models have been invaluable to understand mechanistic concepts of immunotherapy. However, intrinsic characteristics of mouse models create challenges for clinical translation. In particular, preclinical models with intact immune systems that closely mimic the human immune system, display comparable, spontaneous oncogenesis and immune interactions to humans, and that can model key immunotherapeutic outcomes such as efficacy, dose response, and toxicity, will be critical for progress in translational cancer immunotherapy research.

In this review, we will highlight why the study of spontaneous cancers in companion animal dogs is an attractive model for overcoming obstacles in cancer immunotherapy research. First, cancer is a leading cause of death in dogs, as it is for humans. Consequently, the use of companion dogs for the study of cancer biology and treatment has been advocated by veterinarians and other translational researchers for more than 50 years [10–16] Secondly, dogs are large, outbred animals that develop cancer spontaneously. The parallel evolutionary history of humans and dogs also has led to greater similarities in the organization of the canine and the human genomes than what is observed between humans and mice, as well as shared exposure to environmental risk factors. Together, these traits appear to make dogs a very attractive translational model for cancer immunotherapy.

### Preclinical models as tools for cancer immunotherapy

For over 100 years, preclinical animal models have been the foundation for the development of novel cancer therapies. Historically, this foundation has relied on mouse models, and there is no question that these models remain fundamentally important today [17, 18]. The vast majority of current in vivo cancer biology studies use inbred laboratory mice, and the pre-eminence of rodent studies in cancer experimental therapeutics is unlikely to be displaced in the near future. In particular, genetically engineered mice (GEM) have been especially informative regarding mechanisms of oncogenesis and the identification of novel targets for therapy. However, practical considerations limit the number of genes and mutations that can be effectively studied in GEM models. Furthermore, GEM tumors also might under-represent the heterogeneity and complexity of spontaneous human malignancies, potentially oversimplifying cancer immunotherapy studies where tumor-host interactions, immuno-editing, and immune evasion are key issues [17, 19].

Laboratory mice are generally genetically homogenous, matched for size, age and sex, fed identical diets and housed in specific pathogen-free (SPF) environments. All of these factors are critical for carefully controlled and executed mechanistic studies of promising new anti-cancer agents, but there are increasingly recognized limitations of mouse models. For example, a somewhat controversial study by Seok et al. simultaneously analyzed the genetic changes occurring in humans and mice following inflammatory insults such as burns, trauma and endotoxemia. Although there was high genomic similarity after different inflammatory conditions among different human subjects, a surprisingly poor correlation of genomic changes was observed between humans and mice [20]. Consequently, although there have been subsequent reports challenging these findings, this study was an important statement on the limitations of mouse models for the study of human disease and underscored the potential for differences in mouse and human biology to confound results. Moreover, it is increasingly recognized that studies using young, sex-matched, typically female mice often fail to accurately represent the older, obese and heterogeneous human population that develops cancer [17, 21]. These are important considerations given that only 11% of oncology drugs which work in mice are ever approved for human use [22, 23].

There are other aspects of the controlled environment in which inbred mice are housed that can create an inaccurate representation of the human disease. For example, differences of environmental/microbiome factors have recently been implicated in response to cancer treatments, including immunotherapy. In fact, recent studies demonstrated that differences in the gut microbiota of mice raised in SPF environments at different research institutions affect both tumor growth rates and responses to immunotherapy [24–26]. In contrast to mice, pet dogs seem to share many features of the human microbiome. Song et al. analyzed the effects of co-habitation among related and non-related children and adults as well as dogs living in the same household on the range of microorganisms found on the skin and the intestines. The authors observed that co-habitation, including dog-human co-habitation, likely from frequent contact, was the strongest predictor of similarities in microbiota with the skin showing the highest concordance [27]. Microbiome studies in dogs have also demonstrated that the resident microbiota is an important driver of host immunity and inflammation [28]. Although detailed studies of the microbiome in dogs undergoing cancer treatment or immunotherapy have not been performed, the studies to date highlight the potential for dog microbiome studies to be generalizable to and representative of the broader human population.

A greater challenge for translational immunotherapy is that many laboratory models now utilize immunocompromised mice as hosts for human tumor-immune cell xenografts and patient-derived xenografts (PDX). As the constituent elements of the immune system are not completely represented in these animals, the models fail to represent the full complexity of tumor-host interactions. Humanized mice that recapitulate components of the human hematopoietic and immune system circumvent some of the concerns associated with studies using immunodeficient mice and therefore represent potential translational tools [23, 29]. However, these models are costly, technically complicated (MHC-typing or use of transgenic mice are necessitated), and ultimately still lack critical functional components of the human immune system, which limits their ability to truly mimic the context in which spontaneous human cancers develop [19].

Moreover, despite the increasing sophistication of humanized mouse models (of which HLA- and human cytokine transgenic mice are available) as well as other key advances in mouse cancer modeling, pre-clinical mouse models are still limited by artificial factors such as the SPF environment in which inbred mice are housed, leading to unrepresentative environmental/micro-environmental factors, including the microbiome. Chronic viral infections (such as Epstein-Barr virus and cytomegalovirus) that heavily shape the human immune system repertoire are also not present. Recent studies have demonstrated that mice raised in SPF environments in different institutions will manifest distinct microbiota which affects rates of tumor growth and immunotherapy responses, raising key questions about host-tumor interactions in the response to immunotherapy [24–26].

Of all animal models, non-human primates (NHP) are the most similar to humans in their genetic composition [30]. Yet, interestingly, but for unclear reasons, NHP raised in conventional primate centers have a low incidence of spontaneous cancer (while cancer incidence and prevalence for NHP in the wild is unknown) [31]. As a result, NHP have proven less useful as tumor models. Furthermore, the high cost of breeding and housing NHP as well as ethical issues are important barriers to their use as a preclinical model [32].

The limitations of conventional mouse models underscore the need for novel approaches to understand the spectrum of responses, both in terms of efficacy and toxicity, which are observed in human cancer patients that receive immunologic and biological therapies. We propose that the dog model provides a critical link in pre-clinical studies since dogs are large, outbred, immunocompetent animals that develop spontaneous tumors. The principal advantages and disadvantages of the various pre-clinical animal models for cancer immunotherapy studies are summarized in Table 1.

**Table 1:**
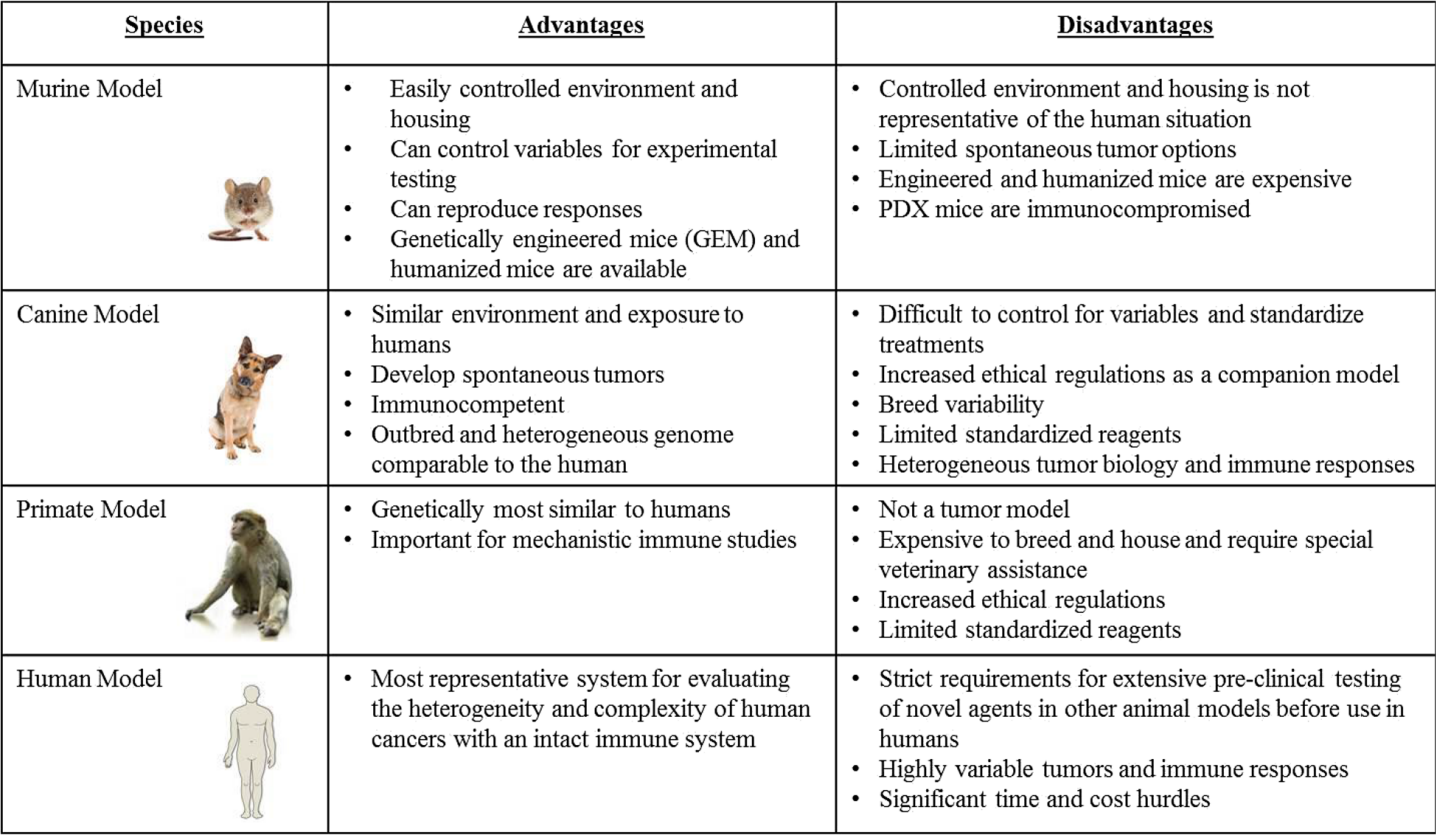
Summary of Commonly Used Immunotherapy Models/Systems

### Canine cancers

Although rigorous epidemiological data are not available for companion animals, current estimates suggest that approximately 2–4,000,000 dogs in the US are diagnosed with cancer annually [33, 34]. Overall, the best available data estimate that approximately one in four dogs in the US will be diagnosed with cancer, which (if confirmed) would translate to an incidence of canine cancers per year strikingly greater (5300 cases/ 100,000 dogs) than that of humans (500 cases/ 100,000 persons) [33, 34]. With growing cancer diagnoses in both human and canine populations, spontaneous cancers in dogs make them ideal for the study of cancer biology and immunotherapy, especially since pet owners are highly driven to seek out novel treatments for their companion animals. Moreover, with the decoding of the canine genome, important similarities between dog and human oncogenesis, including specific cancer-associated genes such as BRAFV600E, p53, Bcr-Abl, and c-kit have been recognized [35–37]. In osteosarcoma (OSA), for example, gene expression profiling has demonstrated remarkable homology between canine and human forms of the disease, reinforcing the shared biology between dog and human [38, 39].

In addition, cancers that develop in dogs show the same complex interplay of genetics, age, and environmental exposures as in humans [12, 33, 40, 41], and these similarities are stronger between humans and dogs than they are between humans and mice [30, 33, 42–44]. As with humans, cancer incidence in dogs is associated with increasing age, although certain cancers do display distinct epidemiological patterns between dogs and people [45, 46]. For example, in humans, OSA is markedly more common in children and adolescents, whereas the diagnosis peaks in middle age to older dogs between 7 and 9 years [47]. Interestingly, the risk of OSA in dogs also increases with increasing body weight and is specifically associated with large and giant breeds such as Great Danes, Saint Bernards and Irish wolfhounds [48]. Somewhat paradoxically, although outbred at the species level, the genetic diversity of dogs is also quite restricted at the breed level [44]. This artificial genetic selection has clearly placed different breeds at elevated risk for certain malignancies as shown in Table 2 [34, 49].

**Table 2:** Common Canine Cancers with Key Demographic Features

Cancer	Incidence	Age of Onset (years)	Location	Breeds at Elevated Risk^a^
Gliomas	2–3X more common in dogs than people	Variable, majority > 6	Intracranial	Boxers, bulldogs, and terriers
Lymphoma	~250,000 new cases per year (2/3 B cell lymphoma)	7–10	Multicentric/external lymph nodes	Golden Retriever, Boxers, Bullmastiffs, Basset Hounds, Saint Bernards, Scottish Terriers, Airedales, Bulldogs
Mammary Carcinoma	Uncommon in spayed female dogs, 10–15% of unspayed females	10–11	Breast tissue	Poodles, Dachshunds, and Spaniels
Melanoma	5–10% of dog cancer deaths	≥10	Mouth, toenail bed, and skin	Terriers, Retrievers, Schnauzers, and Chow Chows
Osteosarcoma	50,000–75,000 cases per year (~75X more common in dogs)	Bimodal, highest peak at age 7–10	Axial and appendicular skeleton	Labradors, Golden Retrievers, German Shepherds, Dobermans, Weimeraners, Boxers, Great Danes, Rottweilers, Irish Wolfhounds
Soft Tissue Sarcoma (e.g. Fibrosarcoma, Myxosarcoma, Hemangiosarcoma)	~10X more common in dogs than humans	All ages	Soft tissues	Labrador Retriever, Golden Retriever, German Shepherd, Bernese Mountain Dog

^a^Data on breed predisposition of specific cancers are potentially subject to reporting bias given differences in breed popularity and differences in how owners may seek veterinary care

Although dogs develop cancers from tissues throughout the body similar to humans, the incidence and prevalence of different tumor types show other notable differences from human counterparts. For example, while OSA, malignant mast cell tumors, and hemangiosarcoma are some of the most common malignancies in dogs, these cancers are uncommon in humans [34, 47]. Similarly, while non-Hodgkin’s lymphoma (NHL) occurs in humans with an estimated incidence rate of 19.6 per 100,000 people, NHL occurs with an even greater frequency in dogs (in 2014, approximately 250,000 cases of lymphoma were diagnosed in dogs versus approximately 71,000 cases in humans) [50–54]. Conversely, human colorectal, pancreatic and pulmonary carcinomas that cumulatively account for approximately 40% of estimated cancer deaths in the US, are much less common in dogs with an incidence of less than 1% for each tumor type [55]. Furthermore, there is a markedly lower incidence of canine breast cancer in the US than in other parts of the world or in humans, almost certainly due to the routine practice of spaying dogs in the US [56–59]. Nevertheless, it is important to recognize these differences in the incidence and prevalence of specific cancer types between humans and dogs because these differences impact the translational relevance of canine cancer studies to humans. However, such variations can also be advantageous for clinical translation of novel cancer therapies since the higher incidence of OSA and hemangiosarcoma in canines, for example, can be utilized to obtain clinical data more rapidly than what is achievable in humans with these rare tumors.

Prior to the recent growth in companion animal clinical trials, the laboratory research beagle represented a more standardized way to proceed with experiments in canines. International requirements, particularly in the United Kingdom and in Europe, require toxicology and pharmacology studies in at least two animal species, a rodent and non-rodent, prior to human clinical trials. The non-rodent model is frequently the research beagle, which has been chosen because of its relatively small size as well as its passive nature and affable personality. A single breed also minimizes breed variability that might otherwise exist among studies. Choi et al. and Ikeuchi et al. have provided reference values for hematological, serum biochemical and urological, as well as organ weight parameters to establish a standardized set of normal values, and minimize the use of laboratory canines for baseline studies [60, 61]. However, breed-related variations in hematologic and serum biochemical values have been reported, signaling caution in applying immunologic findings from beagle studies to other dog breeds [62, 63]. Curiously, laboratory beagles anecdotally appear to be less sensitivity to toxicity than most pet dogs [64]. In fact, it has been recommended that when undertaking a Phase I clinical trial of a chemotherapy agent in client-owned dogs, the first dose administered should be 50% of the maximum tolerated dose observed in laboratory beagles because of their apparent favorable toxicity profile. Although the reason for this remains unknown, it does reinforce the concept that data derived from laboratory beagles may not be easily compared to that obtained from client-owned dogs and other breeds.

### Canine clinical oncology

As in humans, cancer treatment of pet dogs relies principally on surgery, chemotherapy and radiation therapy (RT), with several nuances. The decision for dog owners to pursue cancer treatment for their pets may be driven by several considerations including the desire to improve their pet’s quality of life, especially if a cure is not likely, and to prevent or delay recurrence or metastasis. Arguably, the implicit goal of cancer treatment in companion dogs is to elicit maximum benefit while preserving optimal quality of life. Thus, lower doses of chemotherapy agents are frequently delivered to dogs than would be to humans in order to avoid severe toxicity. Another implicit assumption is that cancer care in dogs is more likely to be palliative in intent, rather than curative. Although conventional therapies are typically offered, and ‘standard of care’ approaches are recommended, owners frequently elect experimental therapies for their dogs (including participation in clinical trials) when there is no current ‘standard of care’ for that tumor type, or for altruistic reasons, or due to financial limitations. In veterinary medicine, financial incentives to participate in clinical trials are not considered to be unethical or coercive, since those clinical trials frequently include ‘standard of care’ human cancer therapies as the backbone of therapy in addition to an investigational agent [65].

In addition, there is no established “standard-of-care” for certain types of dog cancer, so these patients are treated using a variety of different approaches based on published literature and clinician preference, and in some cases owners are reluctant to subject their pet dog to potentially morbid procedures such as surgery and RT. Chemotherapy is recommended in the adjuvant setting for highly metastatic tumors such as OSA, or as first-line therapy for systemic cancers such as lymphoma, multiple myeloma, and others. Multi-agent chemotherapy is the recommended treatment for high-grade lymphomas (most commonly diffuse large B-cell lymphoma) in the dog [66]. In addition, since the chimeric mAb rituximab binds an epitope of human CD20 that is not conserved in dogs, numerous canine specific anti-CD20 mAbs are in various stages of development for the study and treatment of dog lymphoid neoplasms [66–70].

As in humans, assessment of response to cancer therapy, especially in the metastatic setting, frequently relies on serial imaging studies. Although the recommended imaging modality will depend on the tumor type and location, in dogs it typically includes thoracic radiographs and/or abdominal ultrasound. While computed tomography (CT) and magnetic resonance imaging are readily available and routinely used in clinical veterinary medicine and positron emission tomography is becoming more accessible, they require general anesthesia in dogs and cost significantly more than these other modalities [71]. Another important aspect of veterinary medicine, particularly for comparative researchers evaluating novel cancer therapies in dogs, is that death in client-owned animals is frequently the result of euthanasia. As such, it is important to recognize the potential for this to bias the results, especially in unblinded and non-randomized studies evaluating survival as the endpoint. Of note, many owners are willing to let their dog undergo a necropsy examination after death or euthanasia. Although this allows for more detailed assessment of tumor responses, immune cell infiltration and potential treatment toxicities, as in humans, successful utilization can be variable and unpredictable.

### Canine immune assays

Although there are many advantages to the canine model, currently a key barrier to detailed mechanistic/correlative studies (outside of the inherent variability and cost associated with clinical monitoring in large numbers) in canine models and clinical trials is the paucity of widely available, standardized, and validated canine reagents for laboratory use. For example, although the fundamental components of the dog immune system have been examined to date, characterization of specific components has been much less detailed. Neonatal and post-natal studies of dogs suggest that canines resemble humans and differ from rodents in that dogs appear to be immunologically competent at, or before, birth [72]. Moreover, similar to humans, the phenotype of lymphocytes in the peripheral blood and tumor microenvironment of dogs with cancer has been linked with prognosis. For example, Estrela-Lima et al. observed that both increased tumor-infiltrating lymphocytes based on phenotypic analysis of single cell suspension of tumor tissue by flow cytometry and increased blood CD4/CD8 ratios were correlated with worse survival in canine mammary cancers [73]. Similarly, elevated Tregs, tumor-associated macrophages, and myeloid-derived suppressor cells, respectively, have been associated with adverse outcome in canine B cell lymphoma and mammary tumors [74–76]. Although these studies reinforce the impression of important homology between dog and human immunobiology, especially in cancer, they also highlight the correlative nature of many canine studies with a notable absence of carefully controlled and functional experiments to satisfy high levels of evidence regarding causation and mechanism. Interestingly, there is evidence for breed effects on immune function which likely relate to the inheritance of particular haplotypes of major histocompatibility complex (MHC) genes and further reinforce the paradox that dogs are a highly outbred species which nevertheless manifest significant effects of genetic inbreeding [77–79].

To address the increasing focus on canine models, researchers and vendors have recently focused on the development and dissemination of commercially available, canine-specific antibodies for basic and translational research, as researchers often rely on human and mouse antibodies which have been validated to be cross-reactive for canine markers. Table 3 shows some cell surface markers that are used to phenotype the various canine immune subsets. Mixed-lymphocyte reactions, co-culture killing assays (Chromium release and/or flow cytometry based), IFN ELISPOT, intracellular cytokine staining, and phagocytic activity of dendritic cells using fluorescent-labeled latex beads are all standard immune functional assays used in canine models [80].

**Table 3:** Phenotype of Canine Immune Subsets

Cell	Positive CD Markers	Negative CD Markers
Helper T cell	CD4,^a^ CD45	CD21
Cytotoxic T cell	CD8, CD45, IFN-γ	CD21
Activated Memory T cell	CD25, CD44, CD45, CD69	CD62L
Regulatory T cell	CD4, CD25, CD45, FoxP3	CD8
B cell	CD22, CD79a, CD45, CD25, MHC2	TCR
Dendritic cell	CD11c, MHC II, CD80, CD14	N/A
Macrophage	MHC II, Mac-3/Lamp2/107b, F4/80, CD11b, CD206	N/A
Natural Killer cell	CD5 dim, CD45, MHC1, MHC2, NKp46	CD5 (after 14 days in culture), CD4, CD21

^a^Possibly unique to canines, CD4 is expressed in granulocytes. Similar to other species, CD4 is expressed in a subset of monocyte-derived cells

Characterization of canine immunoglobulins dates to the work of Johnson et al. in 1967 [16]. While this body of work, and studies that followed, demonstrated that canine IgGs consist of four subclasses, the diverse functions and interactions of canine immunoglobulins with other immune effector cells have remained less characterized [81]. Nevertheless, there has been a longstanding interest in canine specific mAbs, including canine-CD20 targeted antibodies, for therapeutic and diagnostic use [66, 68, 69]. Important for cross-species translational studies, Bergeron et al. demonstrated that canine Fc gamma receptors bind to dog, human, and mouse IgGs, suggesting that a human therapeutic antibody could be effective at stimulating ADCC in a canine therapeutic model, although species differences may result in significant differences in activity as well as eventual neutralization by the host [81]. Speciated antibodies in a dog IgG framework are now routinely developed using the hypervariable regions of the variable antigen-binding domain (Fv) derived from mouse antibodies [81]. Important for immunotherapy studies, expression of checkpoint molecules, including PD-L1, has been observed on several canine tumors including mastocytoma, melanoma, and renal cell carcinoma [82], and elevated CTLA-4 expression using mouse anti-human antibodies has been observed in dog histiocytic sarcoma patients compared to healthy controls [83]. Unfortunately, studies to explore immune checkpoint blockade in dogs will have to wait as reagents against canine PD1, PDL-1, and CTLA-4 are not yet commercially available, nor do they exist in formats that are suitable for clinical translation.

Overall, the major immune subsets have been characterized in dogs, and significant homology with humans has been demonstrated, but notable differences have also been observed. In 1994, an international Canine Leukocyte Antigen Workshop was held, establishing important canine homologues for key leukocyte populations such as CD4, CD8, and CD90 [84]. Subsequently, homologues of CD45R, CD45RA, CD11, and CD62L were also identified. However, despite these advances, characterizing naïve, activated, and memory subsets for T cells and other immune effector cells has remained limited. For example, Isotani et al. characterized canine dendritic cells with morphology and phagocytic function comparable to mouse and human DCs [80]. In addition, the DCs demonstrated expression of MHC class II, CD11c, CD80, and CD86, and these markers have been used to identify canine DCs in other studies [80]. In another important study, Hartley et al. used multiple cross-reactive antibodies including rat anti-human CCR7 and mouse anti-human CD62L to show downregulation of these surface molecules on activated T cells. Based on these data, the authors proposed a schema for distinguishing canine central memory T cells (CCR7^+^CD62L^hi^CTL2.58^−^) from activated T cells (CCR7^−^CD62L^lo^CTL2.58^+^) [85].

Conversely, dog NK cells have proved more difficult to characterize as dogs do not express CD56 and marker systems such as CD3-CD5^dim^ have been used to describe NK cell activity. Overall, although a clear consensus has not yet emerged [86–88], recent work of Foltz et al. and Grondahl-Rosado et al. have independently demonstrated that canine NKp46 expression appears to identify a canine CD3^−^ lymphocyte population with characteristics and cytotoxicity of NK cells [89–91].

### Canine immunotherapy and clinical trial design

The growing application of cancer immunotherapy to veterinary medicine has been discussed in detail by recent comprehensive reviews [92, 93]. Although a frequent approach is to apply novel human anti-cancer agents including immunotherapy to veterinary patients, it is also increasingly common to see novel agents introduced in companion animals first. Oncept® is an example of a xenogeneic cDNA vaccine which contains a plasmid expressing the human tyrosinase enzyme. It is USDA-approved for the treatment of stage II or III canine oral melanoma. (In veterinary medicine, DNA vaccines and live vaccines are approved by the USDA rather than the FDA - http://www.fsis.usda.gov/wps/portal/fsis/topics/regulations/directives/7000-series/mou-fsis-fda). Although the ultimate effectiveness of Oncept® is controversial, the heterogeneity of responses observed with this treatment illustrates an important aspect of dog immunotherapy studies which is both a strength and a weakness of the dog model, namely that responses are variable and multifactorial in etiology. This heterogeneity of responses is therefore predicted to recapitulate the human experience, [94] but in the absence of reliable biomarkers as well as sample size these studies are also limited by their inability to predict who will benefit. Another notable agent which has been approved for a veterinary indication without corresponding approval in humans is oclacitinib (Apoquel®), a Janus Kinase 2 inhibitor, approved for refractory allergic dermatitis in dogs. The mechanisms by which oclacitinib work in dogs is comparable to JAK inhibitors used in humans for myelodysplastic syndrome and rheumatoid arthritis (with similar side effects) [95].

Although the elements of a canine clinical trial are comparable to those of a human clinical trial, including regulatory approval, informed consent, data management, and biostatistical design, the time and resources needed to implement and accrue to canine trials is viewed as substantially less than with human trials. It is currently estimated that researchers are conducting hundreds of clinical trials on dogs and cats across the world (for all indications), and canine clinical trials are generally viewed as 1 to 2 orders of magnitude less expensive than human trials (but correspondingly 10–100 fold more expensive than rodent experiments) [65, 96]. In addition, as with humans, accrual to trials may be unpredictable, and results especially given the prevalence of trials with non-randomized cohorts may be ambiguous. As a result, there is an increasing emphasis to standardize the veterinary clinical trials infrastructure, including the National Cancer Institute-supported Comparative Oncology Trials Consortium and the recently developed American Veterinary Medical Association clinical trials website (https://ebusiness.avma.org/aahsd/study_search.aspx), akin to www.clinicaltrials.gov. It is expected that this growing formalization of infrastructure for companion animal clinical trials will fuel further support among major funding agencies for companion animal studies [97].

### Advances in canine cancer immunotherapy

**Fig. 1:**
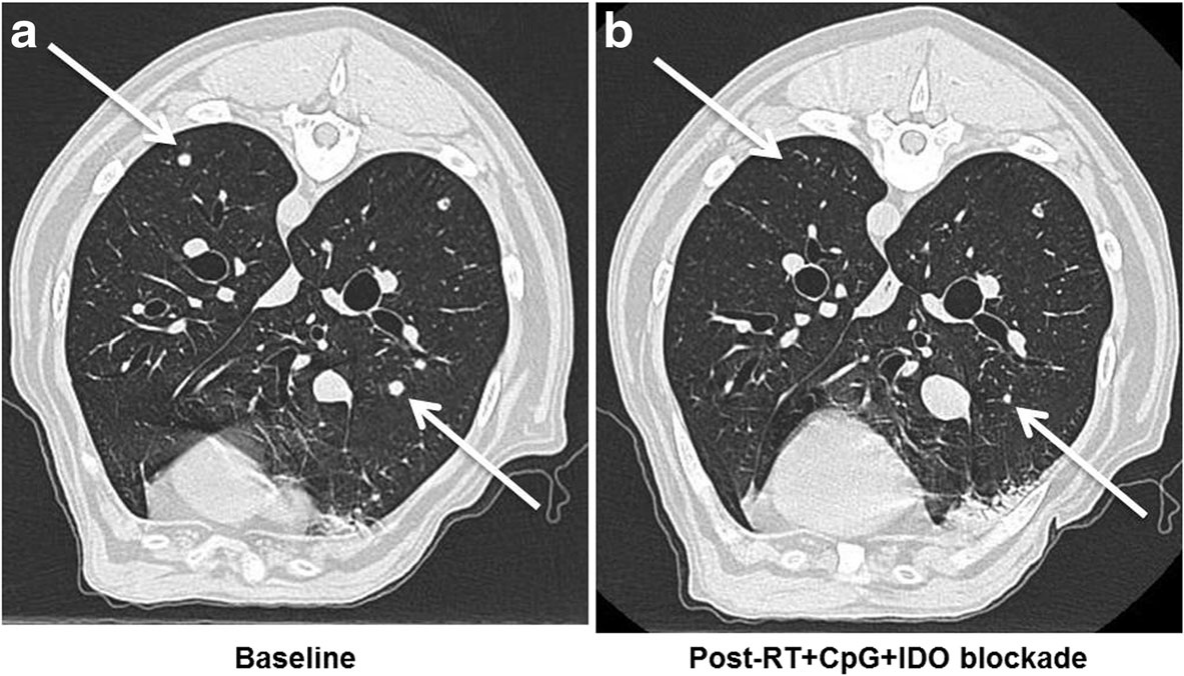
Efficacy of Radiation + CpG + 1MT in a Canine Clinical Trial. Canines with metastatic melanoma and sarcoma were accrued to a pilot clinical trial at the UC Davis School of Veterinary Medicine [98]. **a**. Baseline computed tomography of the thorax demonstrates untreated metastatic lesions in a dog with buccal melanoma. **b**. One month after local RT and intra-lesional CPG to the primary tumor combined with systemic indolamine-2,3 dioxygenase (IDO) inhibition, there is complete regression of some lesions and partial regression of others. Arrows denote index lesions

Paralleling the rapid adoption of immunotherapy in human clinical medicine, immunotherapy in canine veterinary medicine is gaining increasing utilization for both approved and investigational indications. For example, we reported a canine clinical trial in metastatic sarcoma and melanoma testing a novel immunotherapy combination including local radiotherapy (RT), intratumoral CpG oligodeoxynucleotides (immune stimulatory toll-like receptor 9 agonists), and systemic administration of indolamine-2,3 dioxygenase (IDO) blockade with 1-Methyl-Tryptophan (to circumvent immune suppressive pathways) [98]. The premise of this approach was to assess combination therapies involving conventional treatments which are immunostimulatory with strategies to inhibit immunosuppressive pathways [99]. This canine trial was paired with detailed mechanistic studies in murine models which demonstrated that the triple therapy of local RT, intratumoral CpG, and systemic IDO not only reduced intratumoral immune suppression/IDO blockade, but also induced robust systemic anti-tumor effects and tumor regression in five dogs with metastatic melanoma and sarcoma (Fig. 1) [98]. Importantly, a biomarker as shown by reduction in circulating and tumor Tregs was observed in responding but not non-responding dogs. The lack of toxicities associated with the regimen and promising clinical results is leading to human clinical extrapolation.

An example of cell therapy studies under active investigation in canine cancers include the recent CD20-targeting RNA CAR T cells by Panjwani et al. [100]. These authors demonstrated for the first time in dogs that autologous RNA-transfected CAR T cells could be generated, expanded, and administered to a dog with relapsed B cell lymphoma. They observed that treatment was not only well tolerated, but also associated with a reduction in CD20+ B cells in target lymph nodes at 72 h, providing proof-in-concept that CAR therapies can be successfully applied in canine patients with the caveat that more meaningful clinical affects will be contingent on stable CAR expression as long-term engraftment may be an issue. Similarly, Mata et al. tested the ability of genetically modified canine T cells to express a chimeric human HER2-canine TCR CAR T cell [101]. The authors demonstrated successful expansion and activation of the engineered cells which also effectively and selectively killed HER2-positive target cells using in vitro assays. Although not formally tested in a dog trial as yet, the pre-clinical work of Mata et al. on CAR T cells illustrates both the promise and potential barriers of using the canine model. Unique challenges such as reliance on better characterized, more widely available human or mouse-based proteins, cytokines, and transgenes risks the potential for the consequences of xeno-antibody formation [101, 102]. However, given the risk of severe, even life-threatening, adverse events with CAR T cell and other strong immunotherapy regimens, especially when given systemically, clinical trials of these novel therapies in dogs should be helpful to answer key questions about toxicity and efficacy [103].

NK cell immunotherapy approaches are also being assessed in canine models. At our institution, an ongoing phase 2 canine clinical trial is evaluating the intratumoral injection of autologous activated NK cells following palliative RT for appendicular OSA. Treatment consists of palliative RT weekly for 1 month, and following RT, dogs receive two intra-lesional injections of autologous canine NK cells isolated, expanded, and activated ex vivo, supplemented with clinical grade rhIL-2 for in vivo cytokine support. Another issue in dog immunotherapy studies is the difficulty in obtaining cost-effective amounts of recombinant canine cytokines to be given in vivo for such trials. Administration of human cytokines will be eventually neutralized with repeated use. To date, we have accrued eight patients, and preliminary results have been promising showing minimal toxicities, supporting the use of the canine model for the testing of this novel NK approach (manuscript in preparation). Thus, the canine model is well suited for adoptive cellular immunotherapy evaluation.

## Conclusions

There is a growing body of evidence that the spontaneous cancers in dogs represent attractive translational models that bridge mechanistic studies in mice to the heterogeneous human situation where clinical trials are time and resource intensive. Particularly in the burgeoning field of immunotherapy, as a complement to murine studies and human clinical trials, dogs offer an innovative model for translational research, as they present many of the same challenges faced in “scaling up” a therapeutic system dependent on complex interactions between multiple cell types yet under more controlled settings. They also allow for long-term assessment on efficacy and toxicities. Canine clinical trials offer unique access to a rich source of spontaneously occurring, genetically and immunologically diverse cancers with the benefits of reduced time, expense, and regulatory hurdles of a human trial.

Yet, it is important to recognize that there are disadvantages to the canine model, in particular the currently limited canine-specific/cross-reactive reagents and characterized epitopes available for use. Ultimately, as future of cancer therapy appears to increasingly point to immunotherapy, canine clinical/co-clinical trials represent an ideal format for the rapid and clinically relevant translation of novel and high impact immune therapies and immune combination therapies.

## Abbreviations

CAR T cell: Chimeric antigen receptor T cell
CT: Computed tomography
CTAC: Canine thyroid adenocarcinoma
CTLA-4: Cytotoxic T-lymphocyte-associated antigen 4
GEM: Genetically engineered mice
IDO: Indolamine-2,3 dioxygenase
mAb: monoclonal antibody
MHC: Major histocompatibility complex
NHP: Non-human primates
NK: Natural killer
NSCLC: Non-small cell lung cancer
OSA: Osteosarcoma
PD-1: Programmed death receptor-1
PDX: Patient-derived xenografts
RT: Radiotherapy
SCID: Severe combined immunodeficiency
Tregs: Regulatory T cells
US: United States
